# Integrin-Linked Kinase links Dynactin-1/Dynactin-2 with cortical Integrin receptors to orient the mitotic spindle relative to the substratum

**DOI:** 10.1038/srep08389

**Published:** 2015-02-11

**Authors:** Edward James Morris, Kiran Assi, Baljinder Salh, Shoukat Dedhar

**Affiliations:** 1 Department of Integrative Oncology, BC Cancer Research Centre, BC Cancer Agency, Vancouver, British Columbia, Canada V5Z 1L3; 2 Department of Medicine, Vancouver General Hospital Research Pavilion, Vancouver, British Columbia, Canada V5Z 1L8; 3 Department of Biochemistry and Molecular Biology, Life Sciences Institute, University of British Columbia, Vancouver, British Columbia, Canada V6E 4A2

## Abstract

Cells must divide strictly along a plane to form an epithelial layer parallel to the basal lamina. The axis of cell division is primarily governed by the orientation of the mitotic spindle and spindle misorientation pathways have been implicated in cancer initiation. While β1-Integrin and the Dynein/Dynactin complex are known to be involved, the pathways linking these complexes in positioning mitotic spindles relative to the basal cortex and extracellular matrix remain to be elucidated. Here, we show that Integrin-Linked Kinase (ILK) and α-Parvin regulate mitotic spindle orientation by linking Dynactin-1 and Dynactin-2 subunits of the Dynein/Dynactin complex to Integrin receptors at the basal cortex of mitotic cells. ILK and α-Parvin are required for spindle orientation. ILK interacts with Dynactin-1 and Dynactin-2 and ILK siRNA attenuates Dynactin-2 localization to the basal cortex. Furthermore we show that Dynactin-2 can no longer colocalize or interact with Integrins when ILK is absent, suggesting mechanistically that ILK is acting as a linking protein. Finally we demonstrate that spindle orientation and cell proliferation are disrupted in intestinal epithelial cells in vivo using tissue-specific ILK knockout mice. These data demonstrate that ILK is a linker between Integrin receptors and the Dynactin complex to regulate mitotic spindle orientation.

The mitotic spindle of epithelial cells is repositioned relative to the cell body during development and differentiation in response to a variety of intrinsic and extracellular cues^1^. Epithelial cells divide parallel to the underlying substratum to ensure proper space filling and to maintain a single layer of cells. Cells that are able to divide outside of this plane and away from other cells have lost the regular mitotic checkpoint of cell-cell contact inhibition, a hallmark of cancer^2^. During differentiation, the cell division plane changes. When a stem cell differentiates it divides asymmetrically, not in the plane of substratum. One daughter is formed towards the lumen and in a higher plane above the stem cell layer. The other daughter is formed within the basal layer where it maintains its stem cell state^3^. This mitotic spindle orientation is critical for the regulation of cell division and differentiation.

The mitotic spindle has astral microtubules emanating from each centrosome that contact the cell cortex in an end-on alignment. Interactions between the cortex and these astral microtubules position the mitotic spindle relative to the rest of the cell and in relation to external cues such as polarity signals, neighbouring cells and the extracellular matrix^3^,^4^. A microtubule based motor complex, Dynein/Dynactin, generates force against the mitotic spindle to orient it correctly and the orientation of the spindle usually determines the cell division plane^5^,^6^. Several pathways and receptors are known to localize Dynein-based force generation to the cortex during symmetric and asymmetric cell division^7^. During asymmetric cell division, Numa, LGN and Gαi form an apical receptor complex that captures Dynein/Dynactin which acts on the spindle to orient it perpendicular to the substratum^8^,^9^,^10^,^11^,^12^. However, the proteins that localize Dynein relative to the underlying substratum are less clear.

Integrins are transmembrane receptors that interact with extracellular matrix proteins such as Fibronectin and upon binding undergo a conformational change that induces recruitment of Integrin-interacting partners to the cortex^13^. These Integrin binding partners transmit the extracellular signal from Integrin receptors to the cell and cause changes to a variety of processes such as cell survival, migration and proliferation. β1-Integrins are implicated in mitotic spindle orientation relative to the substratum and sense the extracellular matrix so that the cell can divide parallel to it^14^. However, mechanistic details involved in mitotic spindle orientation downstream of β1-Integrin have not been established and significantly, the crucial complex that links β1-Integrin to the force generating machinery is unknown.

Integrin-Linked Kinase (ILK) is a focal adhesion component that links the β1-Integrin receptor to the actin and microtubule cytoskeleton. ILK also plays a role in numerous processes in interphase cells where it acts as a major signalling hub. ILK binds to Integrin receptors at the cell periphery and localizes to focal adhesions where it is involved in transducing Integrin receptor signals into the cell interior^15^,^16^. ILK is involved in epithelial to mesenchymal transition^17^,^18^,^19^, cell migration^20^,^21^,^22^ and integrating signalling pathways^15^,^23^,^24^. In mitosis, ILK associates with distinct centrosomal proteins and maintains proper bipolar spindle morphology through Aurora A, chTOG and RUVBL1^25^. ILK also helps cluster centrosomes in cells that have multiple centrosomes^26^. Since ILK is known to bind to β1-Integrin and also plays a role in mitosis^27^,^28^ as well as in regulating microtubule dynamics^29^ and microtubule polarity^24^, we decided to explore whether ILK might be involved in spindle orientation.

In this report we show that ILK and its binding partner α-Parvin are required for mitotic spindle orientation, most likely by localizing the force generating machinery to Integrin receptors at the basal cell cortex. Our findings suggest a model whereby ILK localizes Dynactin-2 to the basal cortex of mitotic cells and thus acts as a bridge between the extracellular matrix sensing Integrin receptor and the force generating Dynein/Dynactin complex.

## Results

### ILK is required for mitotic spindle orientation

**Figure 1:**
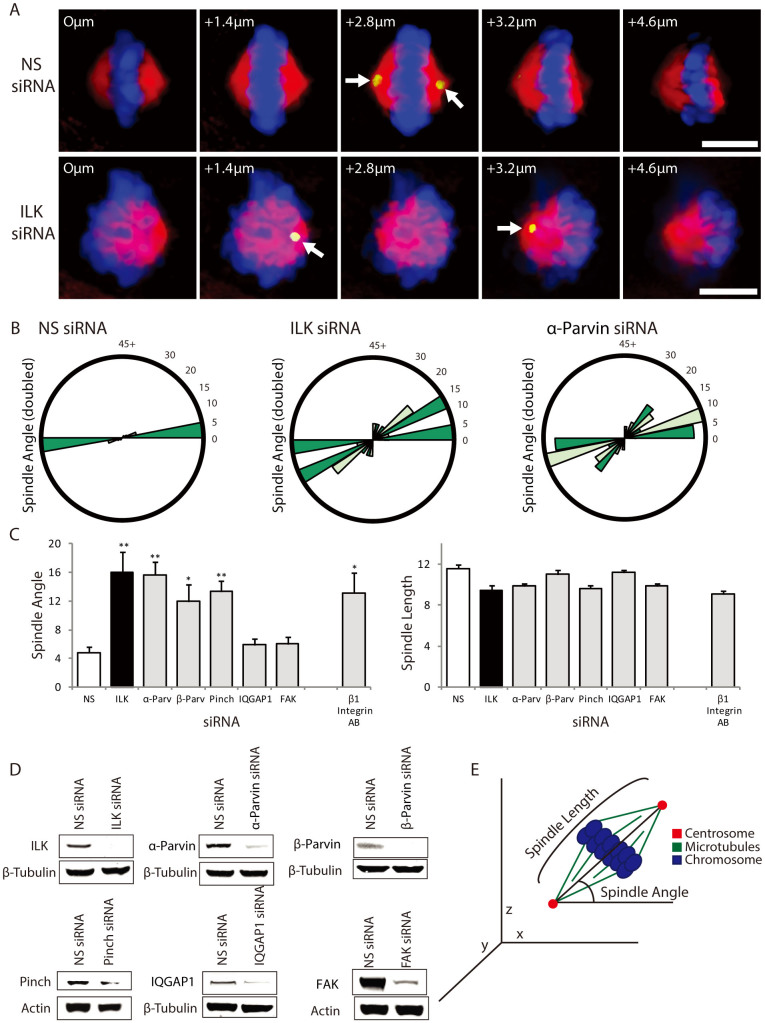
ILK siRNA causes the mitotic spindle to be misoriented. A) Z-sections of non-specific and ILK siRNA transfected metaphase HeLa (Kyoto) cells showing centrosome location (Pericentrin; green/yellow), spindle shape (Tubulin; red) and DNA (Hoechst; blue). Arrows point to centrosomes. Distance from bottom of the cell is denoted on each figure in microns. B) Spindle angle distribution graphs of siRNA treated HeLa cells. C) Measurements of spindle angle along the Z-axis and spindle length in siRNA treated or β1-Integrin inhibitory antibody treated cells. n:30 for each condition. NS denotes non-specific siRNA control. D) Western blots of lysates from siRNA transfected cells demonstrating knock-down of the proteins studied. Western blots were cropped as indicated (black boxes). For all samples, SDS-PAGE and Western blotting were performed under the same experimental conditions. E) Diagram of mitotic spindle angle and length measurements. Scale bar 10 μm. * = p < 0.05; ** = p < 0.01. Error bars represent SEM.

To determine whether ILK has a role in orienting the mitotic spindle, HeLa (Kyoto) cells were treated with siRNA to ILK and the angle of the mitotic spindle was scored. Orientation was scored relative to the coverslip (in the Z axis) and thus relative to the extracellular matrix. Metaphase HeLa cells were stained for centrosomes (Pericentrin; yellow/green), microtubules (α-Tubulin; red) and DNA (Hoechst; blue) and a series of Z-stacks were obtained so that the spindle angle could be measured by assessing centrosomal localization (Figure 1A). HeLa (Kyoto) cells oriented their mitotic spindles parallel to the coverslip when treated with non-specific siRNA (Figure 1A, top panels) but this orientation was more random when cells were treated with ILK siRNA (Figure 1A, bottom panels).

### The IPP complex is required for mitotic spindle orientation

To assess which cellular pool of ILK was involved in controlling mitotic spindle angle, we depleted HeLa cells of individual ILK-interacting proteins using siRNA and then assessed the angle of the mitotic spindle (Figure 1B, 1C, 1D). ILK and α-Parvin increase the average angle of the spindle relative to the substratum when compared to control siRNA treated cells (Figure 1B). ILK knockdown increased the average spindle orientation to 16.0° versus 4.8° for non-specific siRNA. This is similar to other reported spindle angle differences in HeLa cells^30^,^31^. Mitotic spindle lengths were defined as the distance between the two centrosomes and the mitotic spindle angle was defined as the angle that the spindle deviates from parallel to the coverslip in the z-axis and was measured relative to the two centrosomes (Figure 1E). As shown in Figure 1C, ILK, Pinch, α-Parvin and β-Parvin are required for proper mitotic spindle orientation as siRNA to these proteins induce greater randomization of mitotic spindle angle versus non-specific control siRNA. Only cells with normal mitotic spindle morphology were used for determining mitotic spindle angle. ILK, Pinch and the Parvins (α-Parvin, β-Parvin) can form a cohesive unit called the IPP complex^16^. ILK is thought to have roles in the IPP complex at the cell cortex as well as IPP-independent roles at the centrosome^25^,^26^. IQGAP1 interacts with ILK to regulate endosomal fusion^32^ and has also been shown to be involved in microtubule capture in interphase cells^33^ and in cytokinetic function^34^. Focal Adhesion Kinase (FAK) is another Integrin associated protein involved in cytoplasmic signalling of Integrin receptor engagement^35^,^36^. The altered mitotic spindle angle appears to be related to the IPP complex specifically since silencing FAK and IQGAP1 expression did not alter mitotic spindle angle. β1-Integrin is an established extracellular matrix sensor that is required for proper spindle orientation^30^,^37^,^38^,^39^. Inhibition of β1-Integrin with inhibitory antibody treatment also induces randomization of mitotic spindle angle and shows that β1-Integrin attachment to the extracellular matrix is necessary for proper spindle orientation relative to the extracellular matrix (Figure 1C). These data demonstrate that ILK and its associated binding partners, Pinch and Parvin (α and β), but not FAK or IQGAP1 are required for mitotic spindle orientation.

### ILK and α-Parvin localize to the basal cell border of metaphase cells

**Figure 2:**
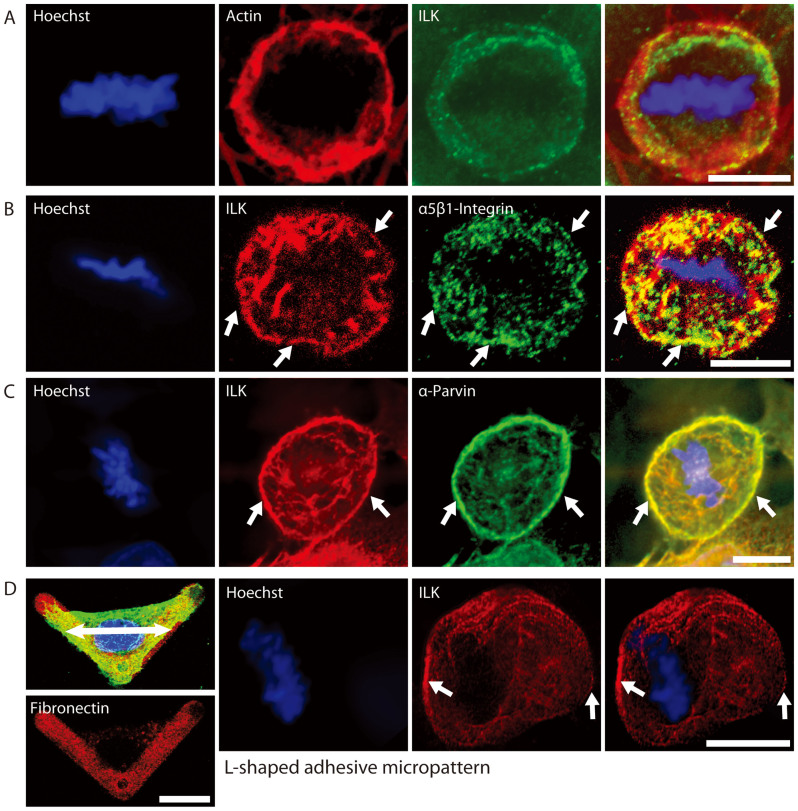
Localization of ILK, Actin, α5β1-Integrin and α-Parvin at the basal lamina of metaphase HeLa (Kyoto) cells. A) ILK localizes to an Actin ring at the bottom of metaphase cells. B) ILK colocalizes with active α5β1-Integrin at the cell edge of metaphase cells (arrows). C) α-Parvin colocalizes with ILK at a ring on the bottom of a metaphase cell (arrows). D) First panels: Interphase HeLa cell adhering to an L-shaped micropattern. ILK (green), Hoechst (blue) and Fibronectin (red). For orientation, the double-headed arrow denotes the direction of tension when the cell enters mitosis. Other panels: ILK and Hoechst staining of metaphase cells grown on L-shaped micropatterned coverslips. ILK aligns with the long axis of the adherent cell, in the direction of tension that aligns the mitotic spindle (arrows). Scale bar 10 μm.

ILK has multiple roles in interphase cells, both at focal adhesions and at the centrosome but this is the first demonstration of a role in mitotic spindle orientation. To gain insight into how ILK regulates mitotic spindle orientation, we assessed the cellular localization of ILK relative to Actin and β1-Integrin in metaphase cells. ILK localizes in the same region as the Actin ring that is found in the cortex at the base of metaphase cells (Figure 2A). ILK binds to β1-Integrin and colocalizes with engaged β1-Integrin in interphase cells where it is involved in transducing signals from focal adhesions^40^. We show that ILK also colocalizes with β1-Integrin at the bottom of metaphase cells in a ring that defines the border of the mitotic cell body (Figure 2B). α-Parvin is tightly associated with ILK in the IPP complex and the two proteins act in concert for various functions^16^,^41^. α-Parvin colocalizes with ILK at the basal cell border, suggesting that the ILK- α-Parvin complex is involved in the spindle orientation pathway we have uncovered and supporting the α-Parvin siRNA knockdown results (Figure 1B).

L-shaped micropatterned coverslips create a defined axis of cell division and are used to predetermine the direction that cells divide. Cells divide along the long axis of the triangle created by the L-shaped micropattern. Multiple pathways have been shown to be involved in aligning the cell relative to the long axis and we decided to determine where ILK localized in this system where the spindle position is predetermined. As shown in Figure 2D, ILK localizes to the poles of the mitotic spindle relative to the metaphase plate (arrows) and to the long axis of the L-shaped micropattern (L-shaped micropattern not shown). At least in the case of L-shaped micropatterns, ILK is positioned to where the spindle pulling forces localize.

On unpatterned surfaces, we noted that untreated HeLa cells occasionally have displaced or misoriented spindles. ILK and α-Parvin, but not Actin, localized asymmetrically at the cell border on the cell side with either the lower centrosome or displaced spindle (Supplemental Figures 1 and 2). One interpretation of this is that the spindle is being pulled on one side, changing the spindle angle and causing misorientation. Since ILK and α-Parvin localize to where the hypothetical pulling occurs and therefore might be involved in force generation, we next decided to see if ILK is involved in localizing known spindle alignment complexes.

### ILK interacts with p50/Dynactin-2 at the cortex to regulate mitotic spindle orientation

**Figure 3:**
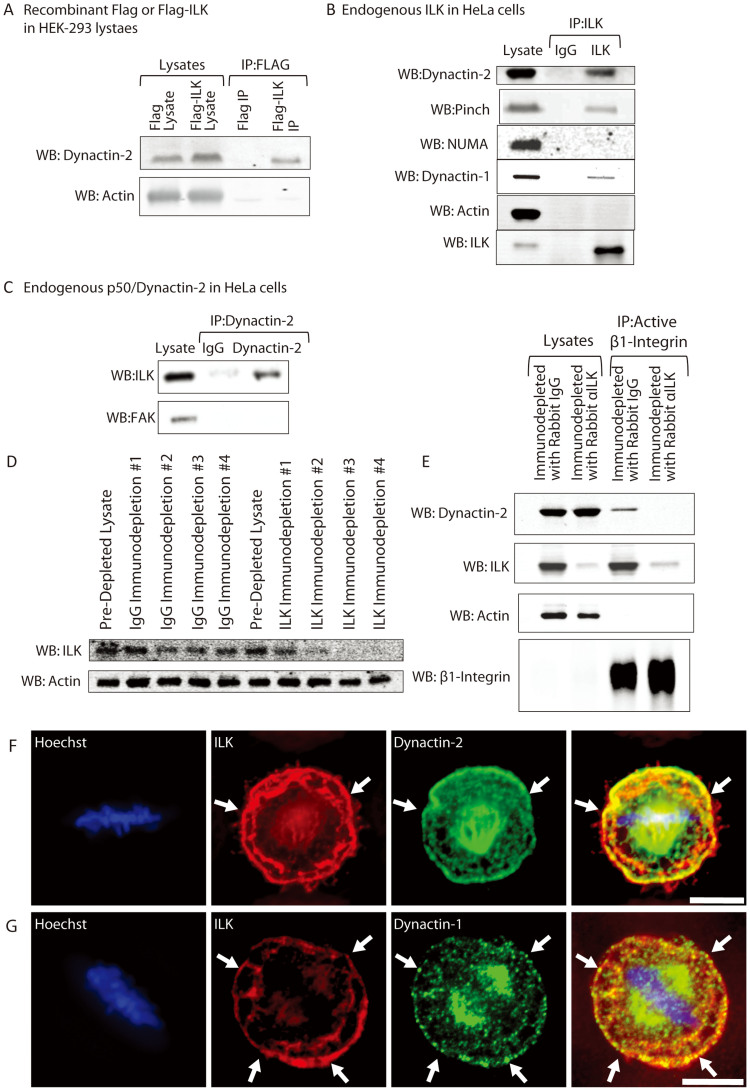
ILK binds to Dynactin-2 and links it to β1-Integrin receptor. Dynactin-2 is part of the linker arm for the molecular motor Dynein. A) Immunoprecipitation of FLAG or FLAG-ILK from HEK-293 cells stably expressing FLAG constructs and western blotted for Dynactin-2 and Actin (loading control). Western blots were cropped as indicated (black boxes). For all samples, SDS-PAGE and Western blotting were performed under the same experimental conditions. B) Immunoprecipitation of IgG (control) or ILK from HeLa cells using endogenous protein and Western blotted for Dynactin-2, Pinch (positive control), NUMA, Dynactin-1, actin (loading control) and ILK (shows amount of ILK immunoprecipitated). Pinch is a known ILK-interacting protein, NUMA is a midcortical anchor for Dynein/Dynactin, Dynactin-1 is another protein in the Dynein/Dynactin complex. Western blots were cropped as indicated (black boxes). For all samples, SDS-PAGE and Western blotting were performed under the same experimental conditions. C) Immunoprecipitation of IgG (control) or Dynactin-2 from HeLa cells using endogenous protein. FAK is another focal adhesion protein and is used as a control. Dynactin-2 staining was not possible due to the rabbit IgG heavy chain from the immunoprecipitation giving strong background signal when stained with rabbit anti-Dynactin-2. Western blots were cropped as indicated (black boxes). For all samples, SDS-PAGE and Western blotting were performed under the same experimental conditions. D) ILK protein levels are reduced in HeLa lysates after 4 rounds of immunodepletion with ILK antibody but not with IgG (control). Actin is a loading control. Western blots were cropped as indicated (black boxes). For all samples, SDS-PAGE and Western blotting were performed under the same experimental conditions. E) β1-Integrin immunoprecipitates Dynactin-2 but not when ILK has been immunodepleted from the cell lysate. Actin staining is a loading control, ILK staining shows ILK is reduced when immunodepleted and is found in β1-Integrin immunoprecipitates. β1-Integrin was detected in the β1-Integrin immunoprecipitates but was not detected in the lysates due to the low level of protein loading used (0.5% of total lysate). Western blots were cropped as indicated (black boxes). For all samples, SDS-PAGE and Western blotting were performed under the same experimental conditions. F) ILK and Dynactin-2 colocalize at the basal cortex but not at the spindle. G) ILK and Dynactin-1 colocalize at the bottom of a metaphase cell. Scale bar 10 μm.

We have shown that ILK is required for mitotic spindle alignment and that ILK is likely acting locally at the basal cell membrane. The localization of ILK relative to the long axis of the L-shaped micropattern suggests that ILK may localize the force generating machinery that positions the mitotic spindle. The mitotic spindle is positioned relative to the rest of the cell by astral microtubules that emanate from spindle centrosomes and span across the cell to the cortex^42^. The primary force generating machinery is the Dynein/Dynactin complex composed of a Dynein molecular motor that uses microtubule shortening to pull on astral microtubules^43^ combined with Dynactin complex proteins that position Dynein and link it to various receptors or cargoes^44^,^45^. If ILK is involved in localizing the force generating machinery then it is possible that ILK interacts with the Dynein/Dynactin complex. We previously performed a proteomics screen to identify ILK-interacting proteins and one of the proteins identified with this tagged recombinant system was p50/Dynactin-2, a subunit of the Dynein/Dynactin complex^46^. Here now, we confirm the interaction of these two proteins through immunoprecipitation of Flag-ILK in HEK-293 cells stably expressing Flag-ILK. As shown in Figure 3A, Dynactin-2 was immunoprecipitated with Flag-ILK but not Flag alone. To determine whether this interaction was physiologically relevant, we immunoprecipitated endogenous ILK from HeLa (Kyoto) cells and western blotted for Dynactin-2. As shown in Figure 3B, Dynactin-2 is enriched in immunopreciptitates carried out using an ILK antibody but not with IgG control antibody. At the midcortex of the cell, Dynein/Dynactin complex interacts with NuMa to orient the mitotic spindle relative to cell-cell contacts. We show ILK does not interact with NuMa through immunoprecipitation, suggesting that the NuMa/LGN/Gαi receptor is separate from ILK. The endogenous interaction between ILK and Dynactin-2 was confirmed by the reciprocal immunoprecipitation where ILK was enriched in anti-Dynactin-2 immunoprecipitate but not in control IgG immunoprecipitate (Figure 3C).

ILK is known to interact with β1-Integrin and one possibility for how ILK is involved in spindle orientation is that it is linking Dynactin-2 to Integrin receptors. If this is the case, depletion of ILK should block any interaction between Dynactin-2 and β1-Integrin. Since ILK siRNA could be acting to interfere with β1-Integrin function in cells, we depleted ILK directly from cell lysates using immunodepletion. ILK was removed from cell lysates using four rounds of anti-ILK antibody directly coupled to Sepharose beads (Figure 3D). β1-Integrin was immunoprecipitated from ILK or control IgG immunodepleted lysates and these immunoprecipitates were probed for Dynactin-2 (Figure 3E). Dynactin-2 is found in β1-Integrin immunoprecipitates but not if ILK was previously immunodepleted. This strongly suggests that ILK is responsible for linking Dynactin-2 to β1-Integrin in HeLa cells.

Since Dynactin-2 and ILK interact the two proteins should also colocalize in cells. Dynactin-2 colocalizes with ILK at the basal cortex (Figure 3F) although ILK does not colocalize with Dynactin-2 at the spindle suggesting that the interaction occurs specifically at the basal cell cortex. ILK also colocalizes with Dynactin-1, another component of the Dynein-Dynactin complex (Figure 3G).

**Figure 4:**
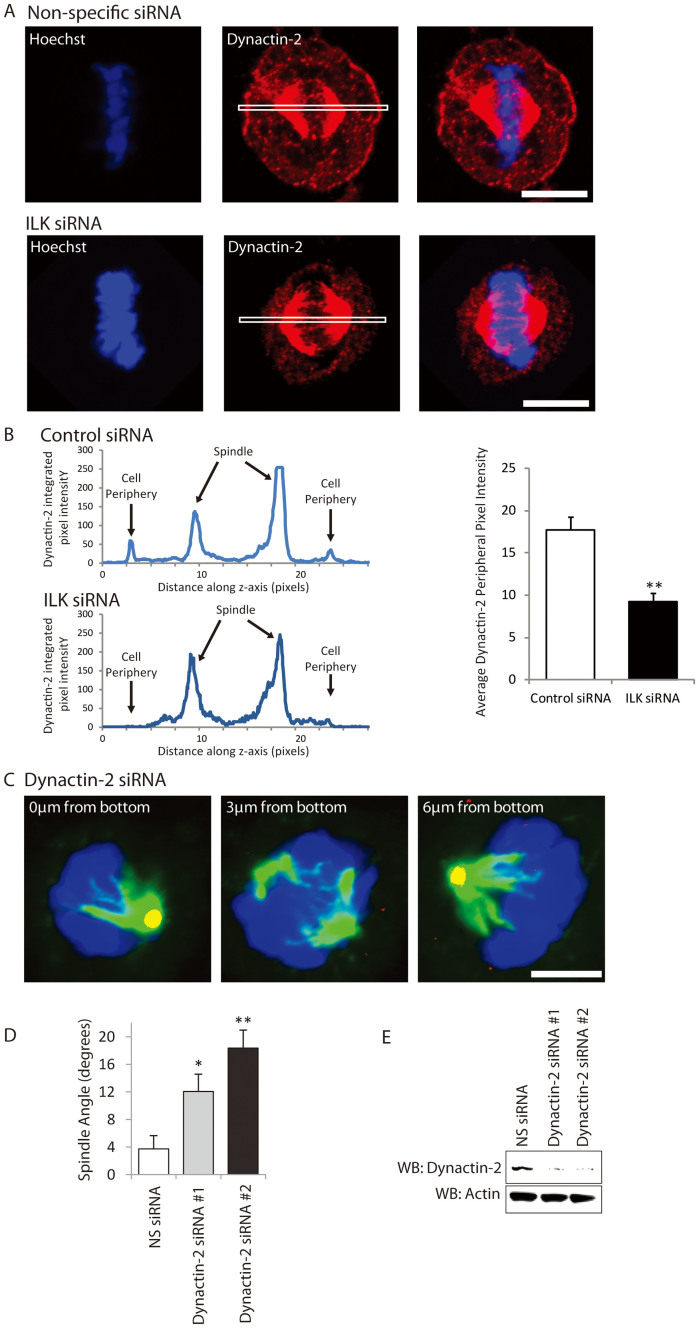
ILK is required for Dynactin-2 cortical localization and Dynactin-2 is required for mitotic spindle orientation. A) Treatment of HeLa cells with ILK siRNA disrupts the punctate distribution of Dynactin-2 at the bottom of metaphase cells, especially towards the edge of the cell (arrows show cell edges). Representative image of 100 cells. B) Line scans of Dynactin-2 integrated pixel intensity in control siRNA (top panel) and ILK siRNA (bottom panel). Areas of the cell scanned are shown by a white box in Figure 4A. Right panel: Quantification of the average Dynactin-2 pixel intensity at the basal periphery of mitotic cells treated with control siRNA or ILK siRNA. C) Z-sections of Dynactin-2 siRNA transfected metaphase cells showing centrosome location (Pericentrin; red/yellow), spindle shape (Tubulin; green) and DNA (Hoechst; blue). Arrows point to centrosomes. D) Quantification of spindle angle in non-specific (NS) and Dynactin-2 siRNA treated metaphase cells. E) Quantification of Dynactin-2 knockdown by Western Blot. Western blots were cropped as indicated (black boxes). For all samples, SDS-PAGE and Western blotting were performed under the same experimental conditions. n:20 for each condition. * = p < 0.05; ** = p < 0.01. Scale bar 10 μm. Error bars represent SEM.

To understand the functional significance of the ILK-Dynactin-2 interaction we examined Dynactin-2 localization in cells treated with ILK siRNA (Figure 4A). Mitotic cells treated with ILK siRNA have substantially less Dynactin-2 at the base of the cell and reduced punctate Dynactin-2 at the edges of the cell (Figure 4B). This suggests that ILK might be involved in recruiting Dynactin-2 to the basal cortex. Dynactin-2 localization to the metaphase spindle appears to be unaffected, suggesting that ILK regulates Dynactin-2 locally and specifically at the basal cortex.

Subunits of the Dynein/Dynactin complex have been previously shown to be involved in mitotic spindle orientation^47^,^48^ but Dynactin-2 has not specifically been implicated in mammalian cells. To determine whether Dynactin-2 is required for spindle orientation we knocked down Dynactin-2 expression with siRNA. HeLa cells treated with siRNA to Dynactin-2 have significantly more random spindle orientation relative to non-specific control treatment (Figure 4C, D, E) and only cells with normal spindle morphology were used to measure mitotic spindle angle (Supplemental Figure 3). These findings suggest that Dynein is using Dynactin-2 as one of the accessory proteins to orient the mitotic spindle and adds significance to the Dynactin-2 interaction with ILK since Dynactin-2 and ILK knock-down have similar effects on the angle of spindle orientation.

Overall, we have shown that ILK and the IPP complex are involved in mitotic spindle orientation, presumably at the basal cortex of the cell where Integrin receptors localize. We have also shown that ILK is likely acting to localize force generating machinery to the basal cortex and that ILK recruits Dynactin-2 to Integrin receptors.

### Mitotic spindles are misoriented in ILK-knockout crypt cells in vivo

**Figure 5:**
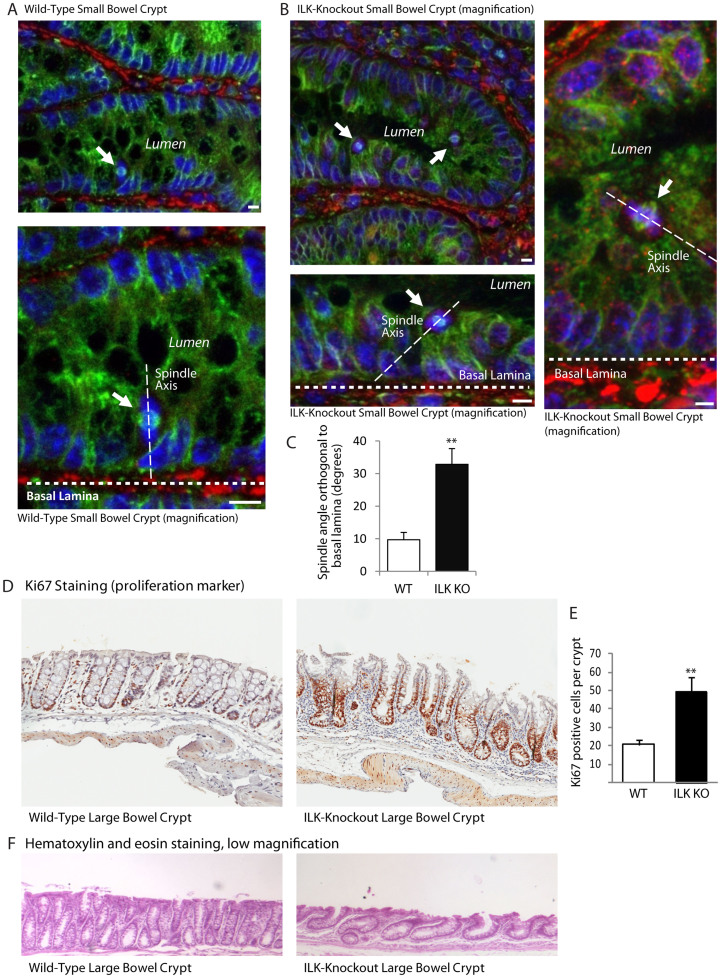
Mice with ILK knocked out in the bowel have misoriented mitotic spindles in their epithelium. A) Immunofluorescence staining of small bowel tissue from wild-type mice showing mitotic spindle are oriented orthogonal to the basal lamina. B) Immunofluorescence staining of small bowel tissue from tissue specific Villin-Cre ILK knockout mice. Mitotic spindles are not aligned relative to the underlying basal lamina. Mitotic spindles (arrows) are aligned orthogonal to the basal lamina (thick white line). Fibronectin (red), α-Tubulin (green), DNA (Hoechst; blue), spindle axis (thin white line). C) Quantification of spindle orientation angle orthogonal to the basal lamina in wild-type and ILK knockout mice. n:20 cells per condition. 4 tissue sections from 2 animals per condition. WT: wild-type. KO: knockout. D) Quantification of proliferation using Ki67 staining in wild-type and ILK knockout large bowel tissue. E) ILK knockout large bowel tissue has greater proliferation relative to wild-type small bowel tissue based on the amount of Ki67 staining. F) Low magnification hematoxylin and eosin stained tissue sections of wild-type and ILK-knockout large bowel. ** = p < 0.01. Scale bar 10 μm. Error bars represent SEM.

To demonstrate that ILK is required for mitotic spindle orientation in vivo, we generated a small-bowel epithelial cell specific conditional knockout of ILK by crossing ILK^flox/flox^ mice^49^ with Villin-Cre mice (Supplemental Figure 4). Tissue sections from wild-type and ILK^flox/flox^-Villin-Cre knock-out mice were either processed for immunofluorescence or hematoxylin and eosin staining. A full description of the conditional ILK knockout mouse line will be published elsewhere. The orientation of the mitotic spindle was assessed relative to the basal lamina in small bowel crypts. Wild-type small bowel epithelial cells had mitotic spindles oriented perpendicular to the basal lamina whereas ILK^flox/flox^-Villin-Cre epithelial cells had more randomized spindle orientations (Figure 5A, B and Supplemental Figure 5). Note that during mitosis in small bowel crypt cells, the cell body containing the mitotic spindle moves in towards the lumen but the mitotic cell remains attached to the substratum^50^. The angle of over 20 spindles was determined in wild-type and ILK knock-out cells and the angles averaged (Figure 5C). The average mitotic spindle angles measured in wild-type sections were very similar to those previously measured for small bowel epithelial cells^50^. In contrast, ILK knock-out mitotic epithelial cells had significantly more random spindle orientation relative to wild-type control epithelial mitotic cells (Wild-type 8.3°; ILK knock-out 32.8°). This demonstrates that ILK is involved in mitotic spindle orientation in vivo.

Cell-cell contacts normally limit cell division but when the axis of mitosis is randomised, division can occur outside of the cell layer. Since ILK^flox/flox^-Villin-Cre epithelial cells can enter mitosis with mitotic spindle not oriented relative to the substratum, we expect that one of the consequences of ILK knockout is that more proliferation is seen and that this proliferation is not restricted to the crypts. As shown in Figure 5D and quantified in Figure 5E, there are many more actively dividing cells in ILK^flox/flox^-Villin-Cre large bowel relative to wild-type. Since aberrant proliferation is a hallmark of early cancer, ILK may play an important role in maintaining homeostasis and limiting early cancer. Indeed, we found that the gross morphology of the large bowel was disrupted with shorter, lopsided and sometimes fused crypts (Figure 5F).

## Discussion

Many studies have previously implicated Integrin receptors and Dynein/Dynactin in spindle orientation. A general consensus is that the Integrin anchor should physically interact with the Dynein/Dynactin motor for the mitotic spindle to orient relative to basal side of the cell as well as the extracellular matrix. However, the molecular pathway linking the two complexes has been completely unknown before now. The most significant and novel finding of this study is that ILK forms a physical bridge between Integrin receptors and Dynactin which gives a mechanism for how the cell is orienting the mitotic spindle relative to the extracellular matrix. This is the first time a link like this has been found and is an important step forward for understanding the many processes where mitotic spindle orientation is essential such as epithelial sheet organization, cancer, stem cells and development^1^. The additional significance of this work is that it demonstrates a new role for ILK in spindle orientation involving Pinch, Parvin and Integrin receptors and shows that ILK plays a role at the basal cortex of cells in mitosis and not just at the centrosome.

Although ILK does play a role at the spindle poles, we have previously shown that its focal adhesion partners Parvin and Pinch are not present at the centrosome and have no role in centrosome maintenance^25^ but in this current study we show Pinch and Parvin are involved in spindle orientation. This suggests that it is the basal cortical ILK/Pinch/Parvin complex that is involved in spindle orientation and not the separate ILK complex at the centrosome. This is supported by several other lines of evidence. First, when ILK was knocked down with siRNA, Dynactin-2 was lost from the basal cortex but not the mitotic spindle which suggests ILK is playing a role at the basal cortex and not at the spindle poles. Second, we show ILK is forming a complex between Integrin and Dynactin-2. Integrin receptors are not at the mitotic spindle and so mechanistically this complex should only function at the basal cortex. Third, we only examined cells with intact mitotic spindles which greatly lessens the likelihood that disrupted centrosomes or spindles are the cause of the mitotic spindle misorientation. In fact, many of the Dynein/Dynactin complex proteins that have been studied have roles in both spindle organization as well as spindle orientation and previous studies of Dynactin/Dynein also ignored cells with aberrant spindles when examining mitotic spindle orientation.

**Figure 6:**
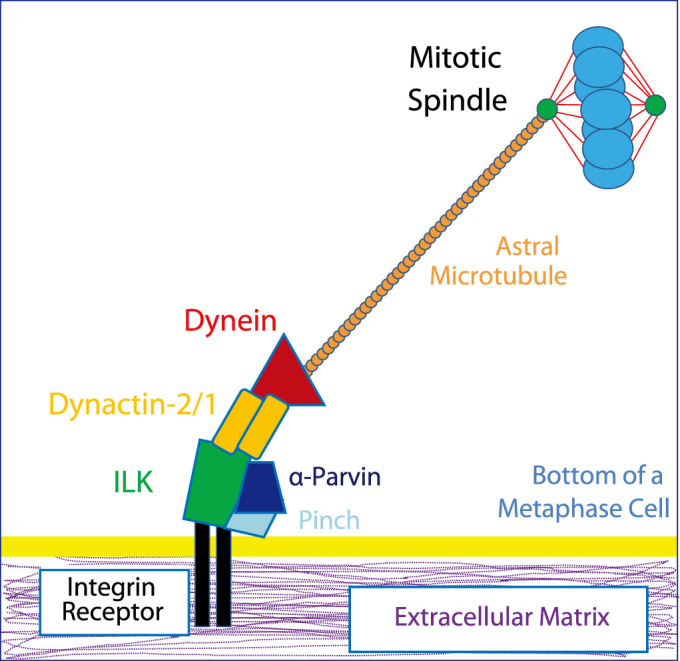
Model for how the spindle is aligned relative to the extracellular matrix. Integrin receptors bind Fibronectin and become activated. This recruits ILK and the IPP complex. ILK binds to Dynactin-2 at the basal cortex which recruits the rest of the force generating machinery that acts on astral microtubules to position the spindle.

Based on the results presented here we suggest a new model for how the mitotic spindle is aligned relative to the extracellular matrix (Figure 6). During mitosis, Integrin receptors bind to the extracellular matrix in a ring that defines the outer edge of the cell body of mitotic cells. ILK and α-Parvin at Integrin attachment sites next recruit Dynactin-2/Dynactin-1 to the basal lamina. Presumably this also recruits the rest of the force generating machinery which acts on astral microtubules to align the mitotic spindle relative to Integrin receptors and the extracellular matrix.

While ILK has a known role at the centrosome in mitotic spindle assembly, this is the first time that ILK and its binding partner α-Parvin have been shown to play a role in mitotic cells at the basal cortex. The significance of this study lies in the elucidation of the molecular basis of cell substrate (ECM) mediated orientation of the mitotic spindle. We have identified a new protein network that potentially links the Integrin receptor matrix sensing apparatus to the Dynein/Dynactin force generating machinery. Other proteins have been shown to link the Dynein/Dynactin complex to the actin cytoskeleton^31^ and a few of the previous models suggested that the mitotic spindle is being aligned relative to actin and not Integrin receptors^51^. Although many proteins have been implicated in coordinating developmental, positional and cell-cell contact signalling with spindle position^51^,^52^,^53^,^54^ and Integrins have been shown to be involved in spindle orientation relative to the extracellular matrix^37^, the involvement of ILK represents for the first time a mechanistic link between Integrin receptors and the Dynein/Dynactin complex.

LGN/NUMA/Gαi is the most researched receptor complex for astral microtubules. LGN and NUMA are scaffolding proteins that link Dynein/Dynactin to the cell cortex via Gαi. Gαi binds to the cell membrane through a myristoylate fatty acid side chain. During symmetric mitosis, this complex is positioned at the lateral sides of the cell so that astral microtubules can be pulled laterally. Lateral positioning is controlled by the kinase aPKC which induces LGN to be excluded from the apical surface of mitotic cells^55^. We did not observe any interaction between NUMA and ILK and also Integrin and NUMA localize to different parts of the cell, suggesting that the Integrin-ILK-Dynactin complex is independent of the LGN/NUMA/Gαi complex. The β1-Integrin/ILK/Dynactin complex that we have uncovered is analogous to the LGN/NUMA/Gαi/Dynactin complex in that there is a receptor (Integrin), a scaffold protein (ILK) and the Dynein/Dynactin complex. Integrin receptors bind directly to the extracellular matrix and they are localized at the basal cortex of the cell and not laterally as for the Gαi receptor. LGN/NUMA/Gαi position the spindle relative to cell-cell contacts and the lateral cell border whereas Integrin receptors are thought to align the spindle relative to the extracellular matrix. During mitosis, Integrin receptors are proposed to act as sensors of the basement membrane although the pathway linking Integrin receptors to the rest of the mitotic cell has been unclear. Based on the data presented in this paper, we propose that Integrin receptors align the metaphase spindle parallel to the basement membrane by binding to ILK which recruits the Dynein/Dynactin complex. As shown in spindle alignment relative to the lateral sides of the cell, we propose that the Dynein/Dynactin complex pulls on astral microtubules emanating from the centrosomes to reposition the spindle relative to cell membrane cues.

Spindle misorientation has been shown to promote tumour formation^56^ and two overlapping mechanisms have been proposed for how this occurs. In epithelial layers, cell-cell contacts inhibit mitosis by physically restraining the cell from expanding and subsequently dividing^2^. When the mitotic spindle becomes misoriented, the cell divides outside of the epithelial layer which means the cell has room to expand and divide into the lumen or into the extracellular matrix. Overgrowth of epithelial cells outside of the epithelial layer is a hallmark of precancerous lesions. The other proposed cancer-causing mechanism involves the role of accurate spindle orientation in the immortal DNA strand hypothesis. Briefly, when stem cells divide asymmetrically they segregate the newly synthesized DNA into the differentiated daughter cell, leaving the daughter stem cell with the unreplicated and therefore error-free original DNA. This reduces mutations in the stem cells and therefore acts as an anti-tumorigenic mechanism^57^. When the mitotic spindle is misoriented, this immortal strand segregation does not occur and cancer is promoted. Our observations demonstrating mitotic spindle misorientation relative to the basement membrane, and simultaneous increased proliferation of epithelial cells in ILK-knockout small bowel tissue, supports the model that spindle misorientation can be cancer-promoting and shows an important role of the Integrin/ILK/Parvin/Dynactin complex in maintaining proper spindle orientation during cell division.

## Methods

### Cell Culture, fixation and transfection

HEK-293 cells stably expressing Flag or Flag-ILK were grown in 10% FBS-DMEM (Gibco BRL) and produced as described previously^46^. HeLa (Kyoto) cells were a kind gift of Prof. Shuh Narumiya (Kyoto University) and were grown in 10% FBS-DMEM. For mitotic spindle orientation measurements, cells were seeded onto acid-washed, Fibronectin-coated coverslips in 0.1% FBS-DMEM and left to adhere for 20 hours before fixation. For spindle angle measurements, coverslips were fixed in 4% paraformaldehyde in PBS then permeabilized in methanol (−20°C). For localization studies, coverslips were fixed using a two step fixation method involving DSP (Dithiobis-Succinimidylpropionate; ProteoChem) fixation then paraformaldehyde fixation using a published protocol^58^. For colocalization of ILK with Parvin and ILK with Dynactin-2, cells were adhered on 10% FBS for greater cell numbers.

HeLa (Kyoto) cells were transfected with siRNA according to the manufacturer's standard protocol using siLentFect transfection reagent (Bio-Rad Laboratories) using 2.5 μL reagent and 7.5 μL siRNA (20 mM) per 3.5 cm well. siRNA duplexes were obtained from Qiagen. Β1-Integrin inhibitory antibody (Millipore clone 6S6) was used at a final concentration of 5 μg per millilitre. Non-specific and ILK siRNA sequences were described previously^25^. The most effective p50/Dynactin-2 siRNAs tested and used for experiments were to p50/Dynactin-2 target sequences CAGCATTGATGAACGGATGAA and CGCCGCCATGGCGGACCCTAA (Qiagen GeneSolution siRNA). Pinch, α-Parvin, β-Parvin, IQGAP1 and FAK siRNA were from Qiagen using recommended siRNA sequences.

L-shaped micropatterned coverslips were purchased from Cytoo and coated in fibronectin. HeLa cells were allowed to adhere to micropatterned coverslips for 10 hours before fixation.

### Antibodies and immunofluorescence microscopy

Mouse anti-ILK (1:800; Sigma; Clone 65.1), rabbit anti-Dynactin-2 (1:200; Novus Biologicals NBP1-85277), mouse anti-α-Tubulin (1:1000; Sigma DM1α), rabbit anti-Pericentrin (1:2000; Abcam 4448), rabbit anti-α-Parvin (1:200; Sigma A1226), Hoechst 3342 (0.2 μg/mL; Life Technologies), rabbit anti-α5β1-Integrin (1:150; Gibco BRL), mouse anti-β1-Integrin (activated state specific; 1:100; Millipore Mab2250), rabbit anti-Fibronectin receptor (1:100; Sigma), Alexa 596-phalloidin (1:50; Life Technologies) were diluted in 5% goat serum (Jackson ImmunoResearch) and 1% casein in PBS and processed using standard techniques. Secondary Alexa 488 and Alexa 596 antibodies were from Life Technologies and used 1:400. Mitotic cells with normal spindle morphology on mounted coverslips were imaged with a laser-scanning confocal microscope (LSM780, Zeiss) and processed using Zeiss software and Image J. For confocal images to show distribution of antibody-labelled proteins at the bottom of the cell, images were deconvolved with ImageJ deconvolution software using the Tikhonov-Miller algorithm. Pictures were colorized and merged using Adobe Photoshop. For measuring spindle angle along the Z-axis, 30 0.2 μm step size images were taken of each cell and the spindle width and angle was calculated using Microsoft Excel. Dynactin-2 levels were quantified by focusing on the maximum intensity image of Dynactin-2 at the basal plane of the cell then scanning images using confocal microscopy. All images were taken with the same settings and processed exactly the same way. Peripheral pixel intensity was quantified in ImageJ.

### Immunoprecipitation, immunodepletion and western blotting

Flag immunoprecipitation was performed as described previously^25^. Endogenous immunoprecipitation was performed in the same manner except cells were lysed in Hepes-Chaps lysis buffer (40 mM Hepes HCl pH 7.4, 120 mM NaCl, 1 mM EDTA, 0.3% Chaps). The p50/Dynactin-2 immunopreciptation was performed in the same manner except cells were first treated with 2 mM DSP and quenched in with 10 mM Tris-HCl prior to lysis. Immunodepletion was performed using four rounds of 1 milligram of antibodies directly coupled to cyanogen bromide Sepharose beads. For immunoprecipitation of active β1-integrin followed by immunodepletion, cells were first crosslinked with 0.8% paraformaldehyde using a published Integrin-specific protocol^59^, lysed in RIPA buffer (50 mM Tris pH 7.4, 1% Triton X-100, 1% sodium deoxycholate, 0.1% sodium dodecyl sulphate) then immunodepleted using either rabbit IgG (control) or rabbit anti-ILK antibody. Immunodepleted lysates were immunoprecipitated with HUTS-21 antibody coupled to cyanogen bromide Sepharose and processed for western blotting. Western blots were imaged with mouse anti-ILK (1:1000; BD Biosciences 611802), rabbit anti-ILK (1:1000; NEB 3856), rabbit anti-dynactin-2 (1:1000; Novus NBP1-85277), mouse anti-PINCH (1:750; Sigma P8996), rabbit anti-α-Parvin (1:1000; Sigma A1226), rabbit anti- β-Parvin (1:1000; ProteinTech 14463-1-AP), mouse anti-FAK (1:1500; Upstate Clone 4.47) and mouse anti-IQGAP1 (1:1000; BD Biosciences 610611), mouse anti-β1-Integrin (1:200; HUTS-21; BD Pharmingen). Secondary antibodies were from Life Technologies and western blots were imaged using the Odyssey infrared scanner (Li-Cor Biosciences).

### Generating Villin-Cre conditional ILK knockout mice and immunohistochemistry

C57BL/6 Villin-Cre mice were obtained from Jackson Laboratory (B6.Cg-Tg(Vil1-cre)1000 Gum/J). Villin has been shown to be expressed primarily in the gut and has been previously used to generate epithelial cell bowel-specific knockout mice^60^. ILK^flox/flox^ mice were a kind gift of Dr. Rene St-Arnaud (McGill University) and have been characterized previously^49^,^61^. The two mouse strains were crossed to obtain ILK knockout specifically in the gut. The offspring were backcrossed 6 times against the Villin-Cre mice to create a uniform background. ILK knockout mice were compared to wild-type ILK^flox/flox^ littermates. ILK protein levels in the small bowel were measured by western blot (Supplemental Figure 4A) or by disassociating the colon epithelial cells with 30 mM EDTA/10% FBS/HBSS then analyzing mRNA expression by qPCR (Supplemental Figure 4B). For qPCR, dissociated epithelial cells were RNA extracted using an RNA extraction kit (Fermentas), reverse transcribed to make cDNA and amplified by PCR in the presence of SYBR Green (Applied Biosciences). Changes in signal during amplification were detected using a Step One Plus qPCR machine (Life Technologies). ILK expression was detected by qPCR using primers 5′- CCAGGTGGCAGAGGTAAGTA-3′ and 5′-CAAGAAATAAGGTGAGCTTCAGAA-3′ and compared relative to Actin mRNA. Details were performed as described previously^61^. Animals were fed chow ad libitum and had liberal access to drinking water. All mouse work was approved by the University of British Columbia Animal Ethics Committee and was conducted in the Jack Bell Research Centre (Vancouver, BC, Canada). The ILK^flox/flox^-Villin-Cre mouse will be fully characterized in a later publication.

Tissue samples from wild-type and ILK^flox/flox^ Villin-Cre mice were formalin-fixed using standard procedures and paraffin-embedded. Tissue section de-waxing, rehydration, antigen retrieval, immunofluorescence and immunohistochemistry were performed as described previously^61^. Rabbit anti-Fibronectin antibody (1:200; Abcam), Hoechst 33342 (0.2 μg/mL, Sigma), mouse anti-Tubulin (1:750; Sigma DM1α) were used as primary antibodies. Mouse anti-Tubulin was detected using biotinylated anti-mouse antibody (3 μg/mL; Life Technologies) and Texas-Red avidin (3 μg/mL; Vector Labs) using the Verastain ABC elite kit (Vector Labs). Rabbit anti-Fibronectin was detected with Cy2 anti-rabbit (1:200; Life Technologies). To score mitotic spindle orientation, mitotic cells were located and measured only when the mitotic spindle was in the plane of focus and fully visible.

## Author Contributions

E.J.M., K.A., B.S. and S.D. designed research; E.J.M. and K.A. performed research; E.J.M., K.A. and S.D. analysed data; and E.J.M. and S.D. wrote the paper.

## Supplementary Material

Supplementary Information Supplemental Information
