# Supplementary material for: Integrin-Linked Kinase links Dynactin-1/Dynactin-2 with cortical Integrin receptors to orient the mitotic spindle relative to the substratum
Source: Sci Rep. 2015 Feb 11;5:8389. doi: 10.1038/srep08389 (PMC4323648; doi:10.1038/srep08389)
Supplement: Supplementary Information — Supplemental Information [file srep08389-s1.pdf]

Supplemental Information

Article Title: Integrin-Linked Kinase links Dynactin-1/Dynactin-2 with cortical Integrin receptors to orient the mitotic spindle relative to the substratum

Edward James Morris, Kiran Assi, Baljinder Salh, Shoukat Dedhar

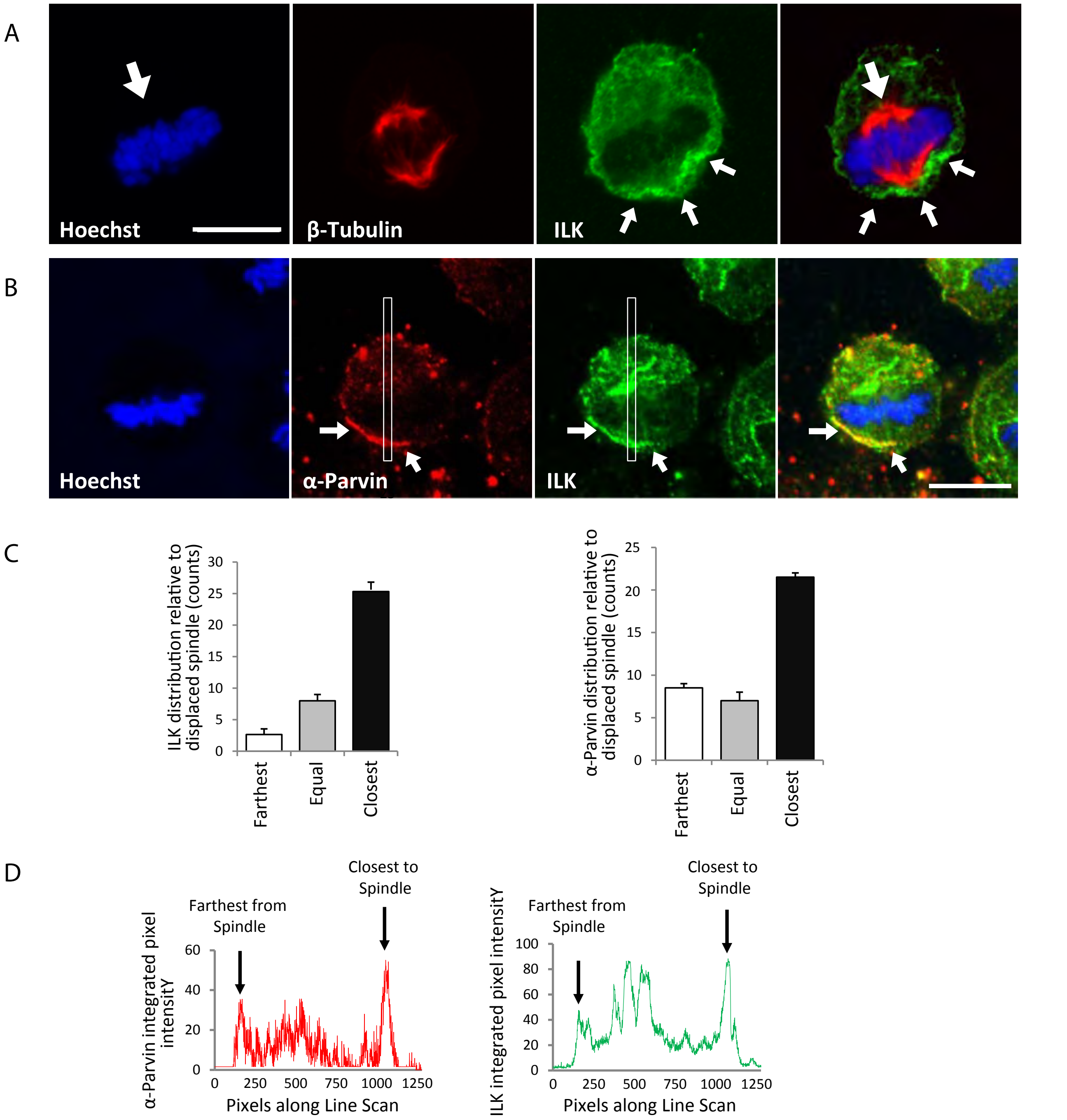

Supplemental Figure 1 Localization of ILK and α-Parvin in metaphase cells with displaced mitotic spindles. A) When the mitotic spindle is misplaced, ILK is asymmetrically distributed towards the side closest to the spindle. Small arrows show the cortical localization of ILK, large arrow shows the displaced metaphase plate at the middle of the mitotic spindle, no longer centered in the middle of the cell. B) α-Parvin colocalizes with ILK on the side next to the displaced spindle (arrows). C) Quantification of spindle position versus polarized ILK and α-Parvin distribution. Images captured from 30 metaphase cells were assessed for ILK and α-Parvin localization in relation to Hoechst (DNA) stain. D) Line scan of α-Parvin and ILK integrated pixel intensies along the white boxes shown in B. Cell borders are denoted by arrows and the peak intensity is highest at the cell edge closest to the displaced spindle. Scale bar 10µm. Error bars represent SEM.

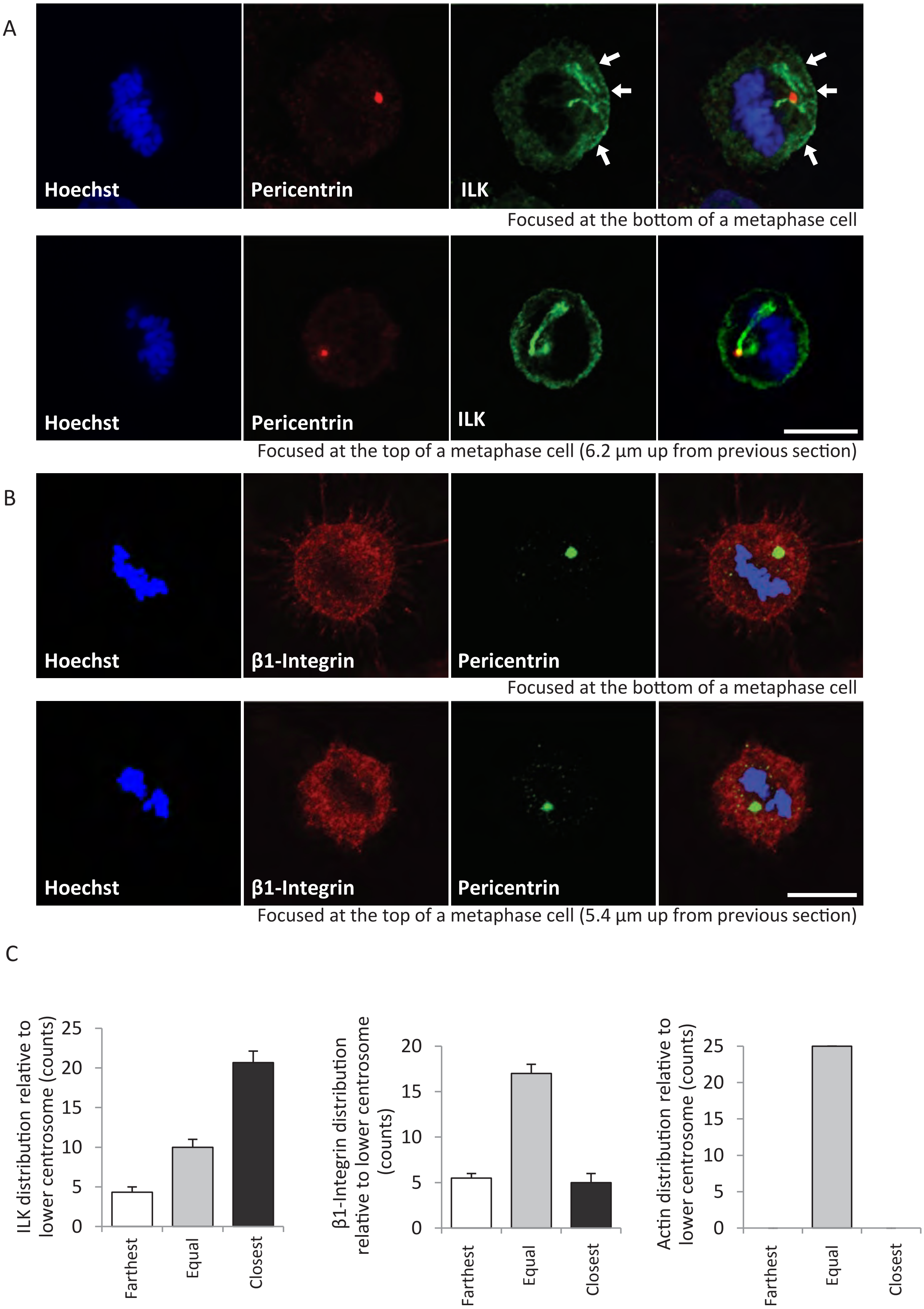

Supplemental Figure 2 When the mitotic spindle is misoriented, ILK is asymmetrically distributed towards the side closest to the lower centrosome. A) ILK distribution relative to centrosome position at the bottom of a metaphase cell with misoriented spindles. ILK is asymmetrically distributed towards the side with the lower centrosome (top panels). Lower panels: ILK distribution relative to centrosome position at the top of a metaphase cell with a misoriented spindle. ILK is not polarized at the top of the cell. B) Active β1-Integrin (Mab 2250) distribution at the bottom (upper panels) and top (lower panels) of a misoriented metaphase cell. Active β1-Integrin is evenly distributed when the mitotic spindle is misoriented. C) Quantification of polarized ILK, active β1-Integrin and actin distribution relative to the lower centrosome position in metaphase cells. ILK is preferentially localized closest to the lower centrosome. n: 25 for each condition. Scale bar 10μm. Error bars represent SEM.

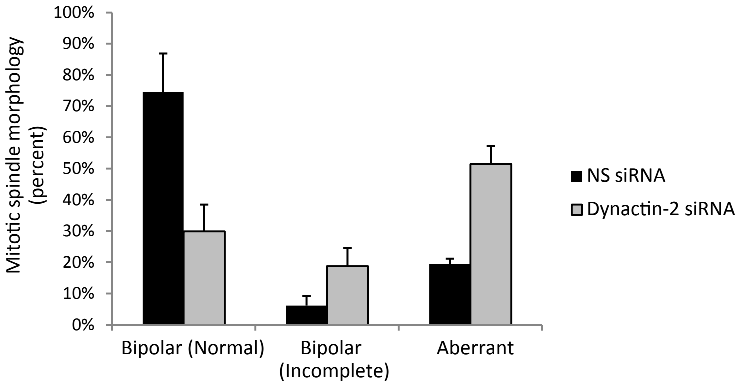

Supplemental Figure 3 Quantification of spindle morphology in control (NS) and Dynactin-2 treated cells. Note that only cells with normal bipolar spindles were used for analysis in the rest of the research. Error bars represent SEM.

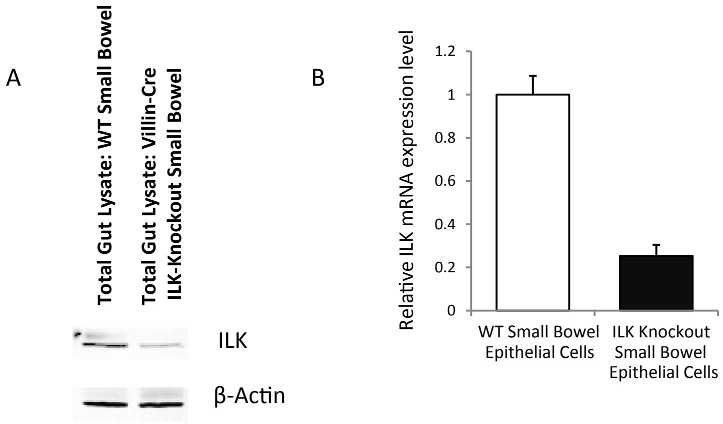

Supplemental Figure 4 ILK-knockout in tissue-specific Villin-Cre conditional knockout mice. A) Western blot of total gut ILK levels in wild-type (WT) and Villin-Cre conditional ILK-knockout mice. B) mRNA levels using qPCR on dissociated epithelial cells. Note: the Villin-Cre conditional knockout gut contains various non-epithelial cells that still express ILK. Error bars represent SEM.

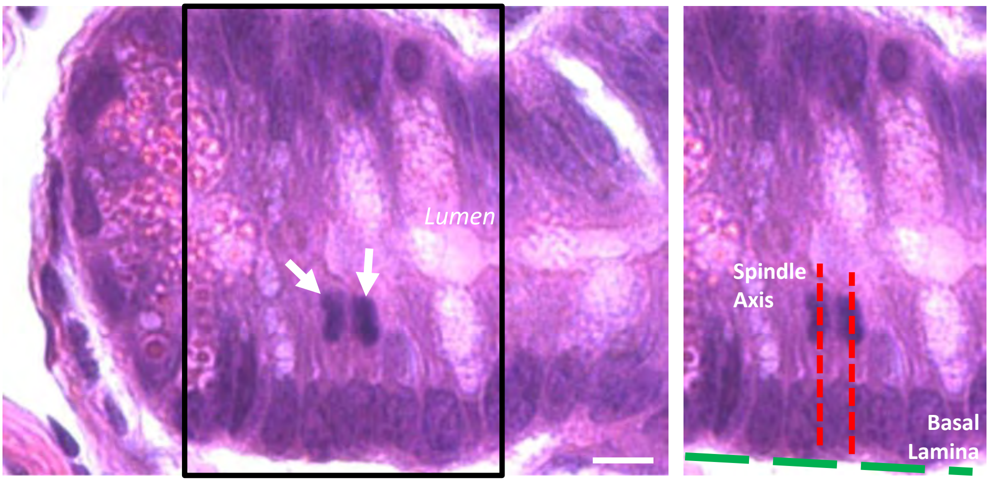

Wild-Type Small Bowel Crypt (magnification)

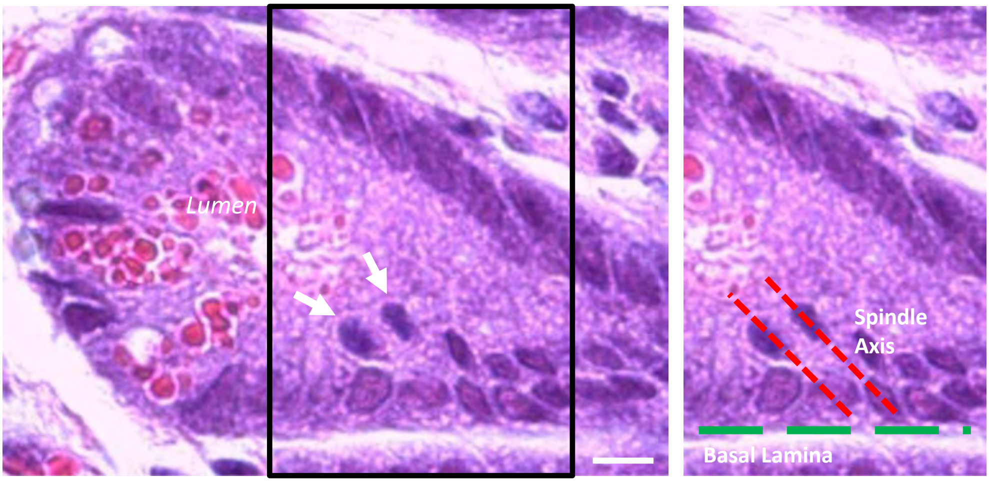

ILK-Knockout Small Bowel Crypt (magnification)

Supplemental Figure 5 Hematoxylin & eosin staining of small bowel tissue from wild-type and intestine-specific knockout mice. Mitotic spindles (arrows) from ILK-knockout cells are no longer alligned relative to the basal lamina (green line). For calculations, dashed red line shows spindle angle versus basal lamina in the right panels. Scale bar 10µm.

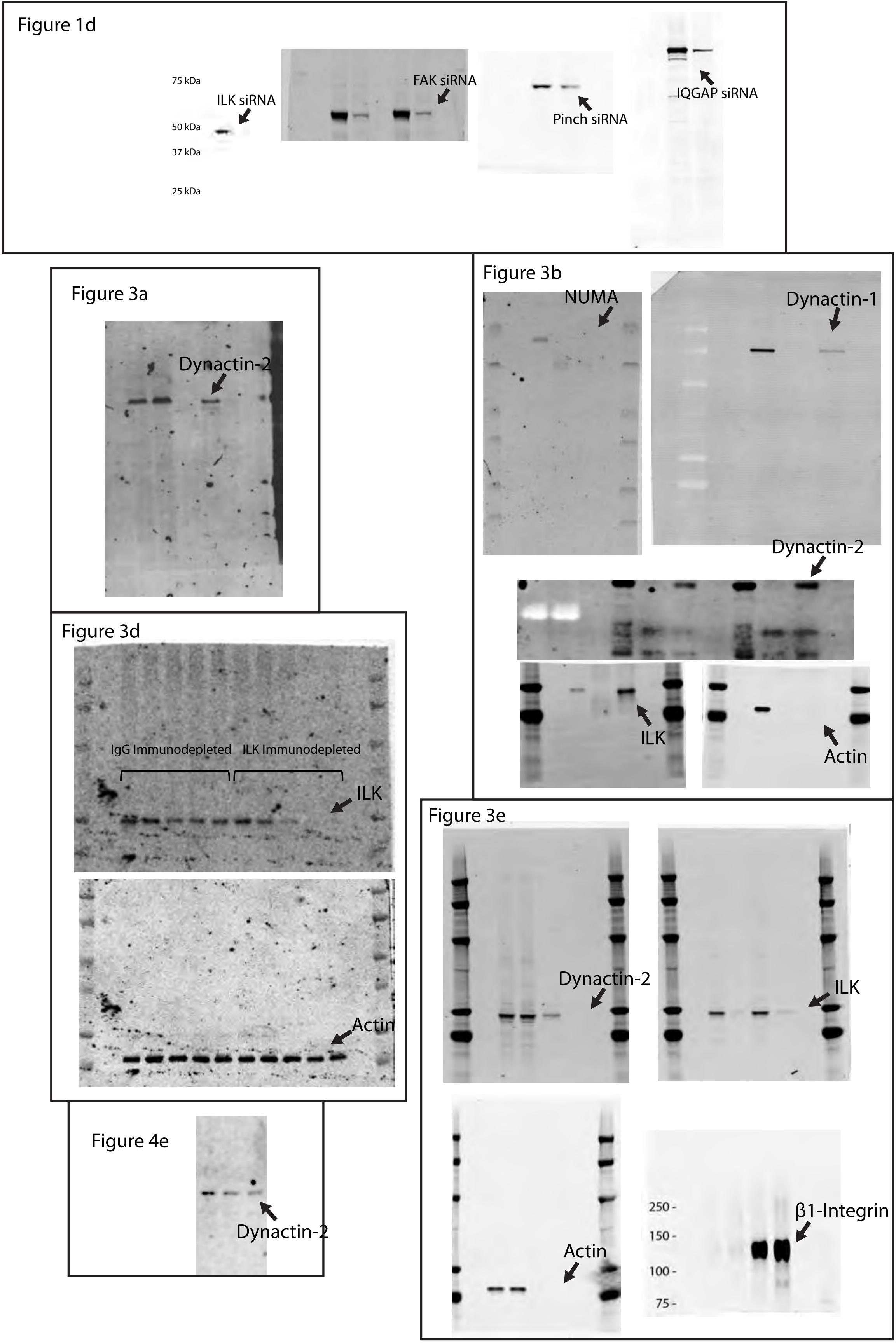

Supplemental Figure 6 Uncropped scans of key western blots.
